# The importance of individual movement and feeding behaviour for long-distance seed dispersal by red deer: a data-driven model

**DOI:** 10.1186/s40462-020-00227-5

**Published:** 2020-10-28

**Authors:** Stephen J. Wright, Marco Heurich, Carsten M. Buchmann, Reinhard Böcker, Frank M. Schurr

**Affiliations:** 1 Institute of Landscape and Plant Ecology, University of Hohenheim, 70599 Stuttgart, Germany; 2 Frankfurt Zoological Society, Bernhard-Grzimek-Allee 1, 60316 Frankfurt, Germany; 3 Bavarian Forest National Park, 94481 Grafenau, Germany; 4 Chair of Wildlife Ecology and Management, Albert-Ludwigs-University of Freiburg, Freiburg im Breisgau, Germany

**Keywords:** Animal behaviour, Animal personalities, *Cervus elaphus*, Endozoochory, Intra-specific variation, Long-distance dispersal, Seed dispersal by animals, Seedling emergence, Seed dispersal

## Abstract

**Background:**

Long-distance seed dispersal (LDD) has strong impacts on the spatiotemporal dynamics of plants. Large animals are important LDD vectors because they regularly transport seeds of many plant species over long distances. While there is now ample evidence that behaviour varies considerably between individual animals, it is not clear to what extent inter-individual variation in behaviour alters seed dispersal by animals.

**Methods:**

We study how inter-individual variation in the movement and feeding behaviour of one of Europe’s largest herbivores (the red deer, *Cervus elaphus*) affects internal seed dispersal (endozoochory) of multiple plant species. We combine movement data of 21 individual deer with measurements of seed loads in the dung of the same individuals and with data on gut passage time. These data serve to parameterize a model of passive dispersal that predicts LDD in three orientations (horizontal as well as upward and downward in elevation).

With this model we investigate to what extent per-seed probabilities of LDD and seed load vary between individuals and throughout the vegetation period (May–December). Subsequently, we test whether per-seed LDD probability and seed load are positively (or negatively) correlated so that more mobile animals disperse more (or less) seeds. Finally, we examine whether non-random associations between per-seed LDD probability and seed load affect the LDD of individual plant species.

**Results:**

The studied deer dispersed viable seeds of at least 62 plant species. Deer individuals varied significantly in per-seed LDD probability and seed loads. However, more mobile animals did not disperse more or less seeds than less mobile ones. Plant species also did not differ significantly in the relationship between per-seed LDD probability and seed load. Yet plant species differed in how their seed load was distributed across deer individuals and in time, and this caused their LDD potential to differ more than twofold. For several plant species, we detected non-random associations between per-seed LDD probability and seed load that generally increased LDD potential.

**Conclusions:**

Inter-individual variation in movement and feeding behaviour means that certain deer are substantially more effective LDD vectors than others. This inter-individual variation reduces the reliability of LDD and increases the sensitivity of LDD to the decline of deer populations. Variation in the dispersal services of individual animals should thus be taken into account in models in order to improve LDD projections.

## Background

For sessile plants, seed dispersal is the “premier spatial demographic process” [1]. It is the movement of seeds away from the parent plant [2, 3] and constitutes a fundamental ecological process relevant at all levels of organisation [1, 3–5]. Long-distance dispersal (LDD) is of particular interest with regards to the preservation of biodiversity, as it plays a pivotal role in linking local populations within a meta-population [3, 6–9], allowing the colonisation of new habitat and facilitating migration and gene flow [3, 7–9]. Colonisation and migration drive latitudinal and elevational range shifts that are particularly important under global climate change.

Endozoochory as a seed dispersal mechanism is well covered in the literature [4, 5, 7, 10–15]. In particular, large and migratory animals are key LDD vectors [4, 12–21]. Ungulates form a large and important group in this regard since they are often the dominant herbivores in a landscape [12], disperse seeds of a variety of plant species [12, 13] and often disperse a large number of seeds (high seed load) [13, 15, 22]. Furthermore, the well documented allometric relationship of body size to gut retention time and movement velocity makes large animals likely effective vectors for LDD [12, 22, 23]. Research into ungulate mediated zoochory has mostly focussed on extrinsic determinants of seed dispersal. These can be broadly divided into being either ‘seed-focussed’ or ‘vector-focussed’. Examples of seed-focussed determinants include seed or plant morphological traits [14, 15], phenology [24, 25], seasonal variation [26] and seed chemistry [27]. Studies of vector-focussed determinants almost always aggregate vectors at the level of species [4, 12, 15, 17, 28, 29], populations [13] or functional groups [16] and seek the drivers of average vector behaviour.

Studies focussing on how inter-individual variation in vector behaviour affects seed dispersal are comparatively rare. Moreover, they use various synonyms for inter-individual variation such as ‘plasticity’ [30], ‘intraspecific trait variability’ (ITV) [31, 32], animal ‘personality’ [33] and ‘behavioural syndrome / type’ [34]. Taking this into account, five potential drivers of inter-individual variation in vector behaviour are identified in the literature, namely sexual dimorphism (physiological differences between the sexes of a species) [35, 36], ontogenetic niche shifts (changes associated with different life stages, especially relating to body size or age) [36, 37], individual specialization (“inter-individual variation in niche that cannot be attributed to age, sex or discrete morphotype”) [32, 36] and behavioural syndromes (aspects of animal personalities including boldness, aggressiveness and curiosity) [34, 36]. Movement and feeding are two key aspects of an individual animal’s behaviour [38–40] and potentially important drivers of variation in LDD services [36].

We studied variation in the movement and feeding behaviour of red deer (*Cervus elaphus*) individuals and the effect this variation has on the LDD services that red deer provide to plant species. We explicitly considered both horizontal and vertical LDD, as it is not only the distance of dispersal, but also its direction which must be taken into account in evaluating its effect [22]. For example, climate change will force some species to migrate either upwards (vertical) or poleward (horizontal) [41, 42] and furthermore, certain plants in mountainous regions may show local adaptation to a specific elevational belt [43].

Studying LDD events is subject to inherent challenges given their rarity [22, 44], the fact that they are often driven by unusual circumstances and/or non-conventional vectors [3, 20, 22, 45] and that they are, by their nature, hard to measure [3, 8, 22, 44]. Most LDD research has thus focussed on identifying and understanding the mechanistic underpinnings of LDD so as to improve the accuracy of predictive models [2, 46].

A general mechanistic model of passive dispersal (dispersal model) [22, 47], defines the dispersal distance of a single seed (*d*) as the product of vector displacement velocity (*v*) and seed passage time (*p*) [22, 48]. Between-seed variation in *p* and *v* are described by two statistical distributions and the product of these two distributions is the distribution of per-seed dispersal distances for a given vector in a given time period. This is known as the vector’s ‘dispersal distance kernel’ [48] and the proportion of this kernel that exceeds a pre-defined long-distance threshold is the per-seed probability of LDD (hereafter LDD probability). The total number of LDD seeds is then the product of LDD probability and the vector’s seed load (Q).

If individual animals differ either in LDD probability or in seed load, they will thus differ in the dispersal service provided to plants. These inter-individual differences will be reinforced if LDD probability and seed load are positively correlated (so that the animals with the highest chance of transporting individual seeds over long distances also transport the most seeds). In contrast, a negative correlation between LDD probability and seed load will tend to equalize the importance of different animal individuals for LDD. Finally, the correlation between LDD probability and seed load may differ between plant species, promoting the LDD of those plants that are preferentially dispersed by the most effective seed-dispersing animals.

We parameterize the general mechanistic dispersal model for individual deer and different plant species by combining individual-specific distributions of displacement distance (*d*) that were determined for GPS-tracked deer, with a distribution of seed passage times (*p*) modelled using experimental data for red deer [23]. We also quantify seed load (Q) from dung collections targeted at the GPS-tracked individuals and a subsequent greenhouse seedling emergence experiment. Our data are collected across an entire vegetation season so as to account for potential temporal effects on movement, feeding behaviour and fruiting phenology. Table 1 summarises how we parameterized the dispersal model.

**Table 1:** Variables used to quantify seed dispersal by red deer with a general mechanistic dispersal model [22]

Variable	How determined?	Variation between deer individuals	Temporal variation
Seed load, *Q*	Germination experiment	Individual-specific	Month-specific
Displacement distance, *d*	Movement trajectories	Individual-specific	Month-specific
Seed passage time, *p*	Feeding experiment [15]	Averaged across deer individuals	–

The table shows how each variable was measured and whether its quantification accounted for variation between deer individuals and months, respectively.

We use the parameterized dispersal model to address the following questions: (1) To what extent does per-seed LDD probability vary between deer individuals and months? (2) To what extent does seed load vary between individuals and months? (3) Are LDD probability and seed load correlated? (4) Do plant species differ in their correlation between LDD probability and seed load? (5) Do non-random associations between LDD probability and seed load affect the LDD potential of individual plant species?

## Methods

### Study site

**Fig. 1:**
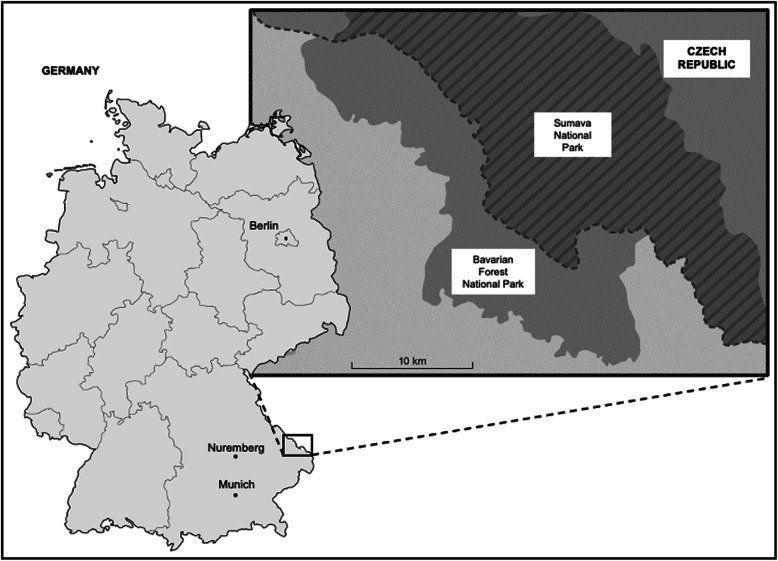
Map showing the location of the study site, the Bavarian Forest National Park, along the German border with the Czech Republic in upper Bavaria. Modified from Wikipedia and Bayerischer Rundfunk [49]

Fieldwork was conducted in the Bavarian Forest National Park (BFNP) in Southeast Germany (Fig. 1) from May to December 2018. Founded in October 1970, it is Germany’s oldest National Park and covers approximately 24,000 ha [50, 51]. Together with the neighbouring Šumava National Park (68,064 ha) in Czech Republic, this area constitutes the biggest contiguous strictly protected forest habitat in Central Europe [52]. A rarity for Europe, the BFNP, with the exception of the buffer zones, is managed according to the maxim of ‘Leave nature to its own devices’. Accordingly, with the exception of path maintenance, no biomass is removed, pests controlled, or diseases combatted within the core zone [50, 51].

The area is mountainous, with elevation ranging from 600 to 1453 m above sea level. The majority of the park is forested (98%). Within the forest, Norway spruce (*Picea abies*) dominates, constituting 70% of the area. The European beech (*Fagus sylvatica*) is also well represented constituting 20% of the forested area. Other species (e.g., *Abies alba*, *Acer pseudoplatanus*, *Fraxinus excelsior*, *Sorbus aucuparia, Betula pendula and Betula pubescens*) are represented in low numbers making up the remaining 10% of forest [50, 51]. Apart from the forest, numerous high-lying meadows (remnants of seasonal pasturing) and raised-bogs of high conservation status also exist in the BFNP and add considerably to its biodiversity and conservation value [53]. Finally, the non-interventionist management approach has resulted in large swathes of forest infested by Spruce bark beetle (*Ips typographus*) with natural succession resulting in repopulation with grasses and herbs attractive to grazers and mixed feeders [51].

### Tracking of deer movement

A total of 21 female, reproductively mature red deer (*Cervus elaphus*) were selected for the study. Adult hinds are known to travel in small family groups with their juvenile offspring [54]. The mean size of the family groups was 1.8 as determined by an aerial survey, in July 2018. This number generally reflected a hind with one calf and was determined using a mixture of infrared and high-resolution natural colour images as per Franke et al. (2012) [55]. The average mass of 37 hinds measured in the study area was 67.1 (± 11.3) kg.

For the purpose of movement tracking, the study animals were fitted with Vertex Plus GPS collars from Vectronic Aerospace GmbH in the winter of 2017 / 2018. Collaring proceeded as follows: Animals were attracted to a corral by food (apple pomace, sugar beets). Within the corral, the deer were led to a capture facility, where the GPS collars were attached without chemical immobilization. A second approach was to tranquillize deer by an immobilization gun, using the Hellabrunner mixture (Ketamin and Xylazine) on sites where they were attracted by food.

The GPS-collars used were fitted with 6-bit GSM and VHF radio communication capacity as well as GPS sensors. Collars were programmed to record one position every hour and transmit these positions via GSM after every seven recordings (i.e. every 7 hours). In the event of a failed transmission (i.e., owing to lack of signal), positions were stored for another 7 hours and sent together with the following batch and so on until transmission was successful. An exception to this schedule was the 1 day a week recording of high-resolution (every 15 min) data. This was done on a rolling day basis (hence if on a Tuesday in 1 week then on a Wednesday the following week). As per usual, locations were transmitted after every seven recordings (in this case every 105 min) with the same rollover rule as per hourly recordings in the event of a failed transmission. For this study we used the movement data collected from May to December 2018.

### Collection of dung samples

A total of 182 pellet groups were collected from 21 individuals over the 8 months from May to December 2018. The focal deer were generally homogenous in that they were all reproductive females of similar size and mass. Juvenile dung is easily distinguishable from that of the mature female by size although there is some risk of erroneous assignment when juvenile males reach the same size as their mother [56].

Data retrieval was done using the GPSPlusX software provided by Vectronic Aerospace. Only positions with an accuracy of 5 m or less (GPS – 3D validated) were used [57] and coordinates were converted to the Universal Transverse Mercator (UTM) standard.

Dung collections were made over two-week sampling periods spread approximately equally (generally the last and first week of every month) between May and December 2018. For each focal deer in each sampling period, we attempted to collect three samples of faecal matter based on the most recent GPS location data available. In the case that multiple pellet groups were clustered in one area, these were treated as a single sample and the remaining two were collected from other spatially distinct (> 30 m removed) deposit sites. Wherever feasible we aimed at collecting samples which were less than 6 hours old (i.e., by responding to GPS fixes that were less than 6 hours old) to ensure freshness and minimize the probability that a sample originated from a different individual. The mean volume of dung samples varied little between deer individuals (172.3 ± 7.4 ml). Following collections, samples were stored in a refrigerator at 4 °C until the end of the two-week fieldwork period and then transported in cooler boxes to the greenhouse.

### Determination of seed-load in the dung (*Q*)

To quantify the abundance of plant species in the dung samples, we monitored seedling emergence from each sample. This measure of seed load thus integrates seed survival in the gut (which may vary between deer individuals). For the experiment, we used a standardised substrate that was prepared by mixing 50 kg of fine quartz sand (0.1–0.5 mm) with 70 l of ‘Hawita Fruhstorfer Typ LD 80’ potting soil containing volcanic clay, peat and long-release fertiliser (pH-value 5.5–6.5). The sand and soil were mixed in a clean cement mixer until homogenous. The substrate was then steamed in a Sterilo soil steamer (Sterilo 7 K 5.4 kW) for 24 h to kill any seeds, fungi or pests. Thereafter we stored it in large plastic containers covered with Organza fabric (29 g/m2) until further use.

Dung samples were gently rinsed with tap water to remove exterior contaminants. The volume of each sample was then determined by measuring the volume of water replaced by each sample. Samples were then mixed in a 1:1 ratio with the aforementioned substrate as well as some water to achieve a homogenously spreadable consistency. Mixing was done using a standard paint-mixing attachment on a variable speed power-drill set at the lowest speed to avoid damaging seeds.

The prepared samples were spread in a layer of approximately 0.5 cm thickness on 60 cm × 40 cm seedling trays filled with the prepared substrate. Where sample size allowed, multiple samples were ‘planted’ in a single tray, with individual samples separated by a clear plastic divider which reached from the bottom of the tray to a height of 10 cm above the substrate. Sample trays were housed in a greenhouse, surrounded by ‘tents’ of organza fabric to avoid seed contamination. Additionally, we established control trays containing identical substrate, but no dung samples to monitor substrate contamination.

As far as possible, greenhouse conditions were maintained at 25 °C during the day and 18 °C at night. Sample trays were hand watered to ensure sufficient and evenly distributed moisture at all times. Artificial lighting (Osram metal halide CCG, Daylight, E40, HQI-BT, 400 W/D PRO, Powerstar) was used to compensate for lack of sunlight during very cloudy periods and in the colder months, thereby ensuring 12 h of growing light per day throughout the experiment. Tray positions were shuffled once a month to avoid micro-climate effects.

As soon as seedlings reached a height of 7–10 cm, they were transplanted into individual pots or seedling trays and allowed to grow until identifiable. Identifications were made using vegetative identification keys [58, 59], and the opinion of an expert on the Central European flora. Each batch of samples was monitored for 3 months and thereafter discarded. Any plants not identifiable at the three-month stage were discarded. Three weedy species (*Cardamine hirsuta*, *Poa annua* and *Erigeron annus*) were found in at least three different control trays and were therefore excluded from the analysis.

Although dung pellet size was initially measured in volume for practical reasons, grams of dry-weight is the more standard unit used in seed dispersal studies. We therefore calculated a conversion factor by taking a random selection of five pellet groups from the months of June, August, September, October and December respectively. For subsamples from each of these 25 pellet groups, we measured the volume and then dried the subsamples at 70 °C for 72 h. The desiccated samples were weighed to within 10 mg, and the measurements were used to calculate an average dry-weight to volume ratio of 0.13 g/ml. It should be noted that dung water content can be subject to substantial seasonal variation (as shown by Edwards 1991 in a South African savannah with strong rainfall seasonality). However, in our study region, which experiences high precipitation year-round, the assumption of temporally-invariant dung water content seems justified. Accordingly, final vector seed load per gram (Q) was calculated as the number of seedlings emerging from the pellet group divided by the pellet-group volume, multiplied by 0.13 g/ml.

### Seed passage time (*p*)

We simulated a distribution of *p* (Fig. S1) based on the statistical model of Picard et al. (2015), [15, 60] who estimated the retention time of six herbaceous plants transported by *Cervus elaphus,* by analysing the results of feeding experiments with a Hierarchical Bayesian statistical model [15, 60]. Their model predicts the probability of excretion in each of 14 time-intervals (up to 1, 3, 6, 9, 12, 15, 18, 21, 24, 30, 36, 42, 48 and 54 h after ingestion). From the posterior parameter distributions of this model, we predicted the posterior mean probability of excretion per time interval for each combination of the six replicate trials of the experiment and six plant species studied. For each of these combinations, we then simulated 100,000 seed passage times to obtain a total of 3,600,000 values of *p* (Fig. S1). We then sampled from this empirical distribution of seed passage times to calculate dispersal distance *d* (see next section).

The plant species used by Picard et al. (2015) represent a wide range of seed size and shape traits, as studying the effect of these traits on retention time was one of the stated objectives of their study [15]. Furthermore, five of the six plant species considered by Picard et al. (2015) were also found in our samples, indicating that their results are appropriate for parameterising our model.

### Seed dispersal distance (*d*)

We generated a distribution of seed dispersal distances (*d*) by drawing 100,000 random endpoint locations (with associated dates and times) from the GPS fixes recorded for each individual deer within the study period (May – December 2018). These points did not necessarily relate to an actual defecation event, but rather a random selection of possible seed deposition sites. For each endpoint, we randomly drew a seed passage time (*p*) from the distribution of simulated seed passage times (Fig. S1). This served to calculate the simulated start time of dispersal and the associated start point on the individuals’ movement trajectory. The straight-line horizontal or vertical displacement (*d*) was then calculated as the difference between the start and end point of the movement trajectory.

**Fig. 2:**
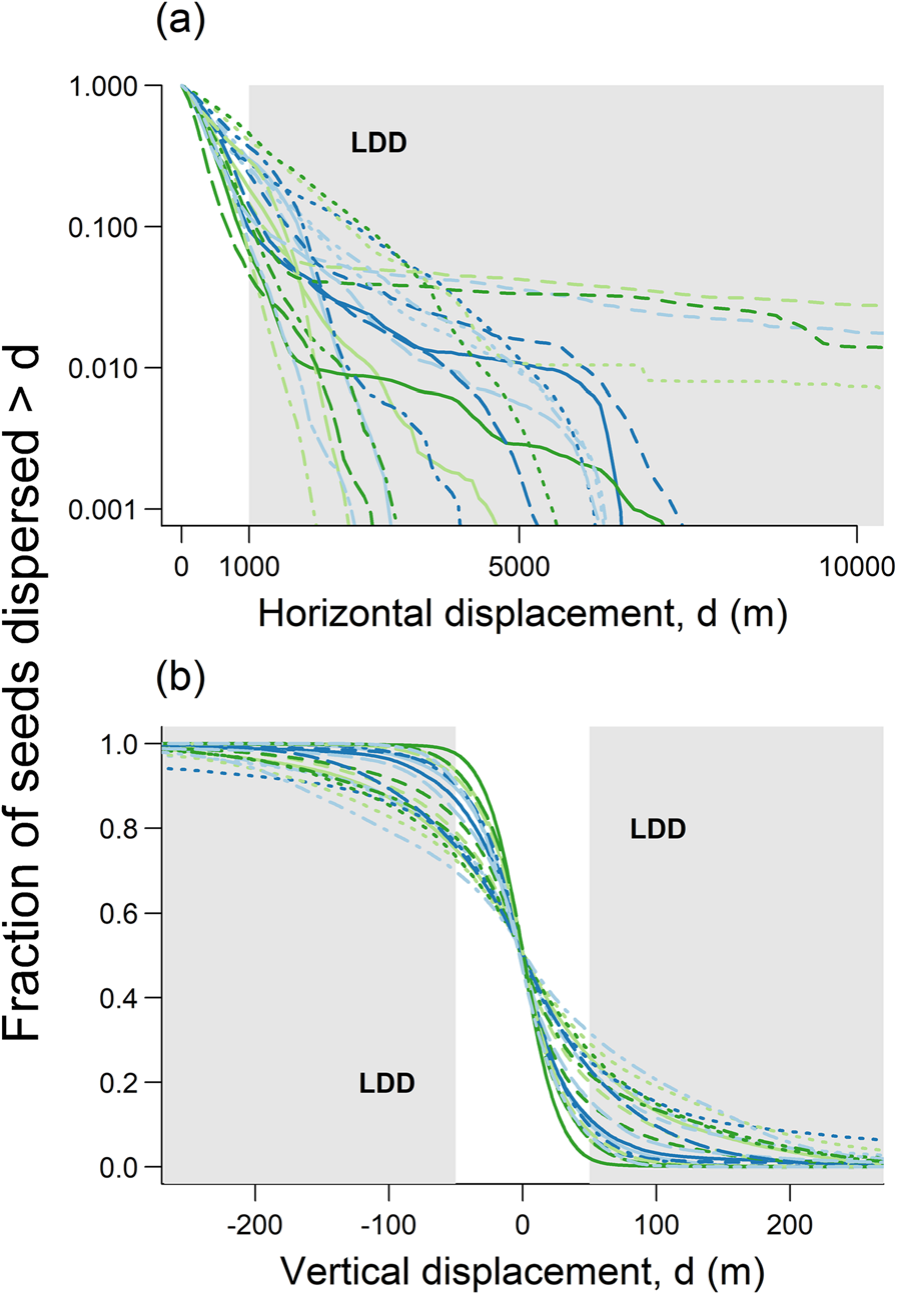
The distribution of horizontal (a) and vertical (b) seed dispersal distances simulated for 21 red deer individuals (indicated by different line types and colours). Light grey areas indicate long-distance seed dispersal (LDD) events exceeding 1000 m horizontal and 50 m absolute vertical displacement, respectively. Note that the y-axis in panel (a) has a logarithmic scaling

Per-seed LDD probability for a given deer individual and month was calculated as the proportion of seed dispersed beyond a threshold distance. We defined horizontal LDD as any horizontal displacement beyond 1000 m (Fig. 2a). We chose a horizontal LDD threshold of 1000 m since it is beyond the extent of most plant populations, exceeds the scale over which most non-vertebrate dispersal vectors typically disperse seeds [61] and falls within the range of scales over which large animals are expected to be relevant LDD vectors [48]. For vertical (upward or downward) LDD, we chose a threshold of 50 m (Fig. 2b) which on average corresponds to a temperature change of ~ 0.3 °C and a latitudinal displacement of 50 km [62]. Everything else being equal, upward seed dispersal of 50 m thus markedly reduces climate change impacts whereas downward dispersal of the same magnitude reinforces these impacts. Note that we used an absolute definition of LDD (as the proportion of dispersal events exceeding a threshold) rather than a proportional definition (as some high quantile of dispersal distance) [22]. This is because absolute LDD definitions are directly related to the environmental context of dispersal, whereas proportional definitions capture extreme, rather than long, dispersal events. The latter can be particularly misleading for efficient LDD vectors that regularly transport many seeds over long distances [8, 22, 42, 48].

### Statistical analyses

To address questions 1 and 2, we quantified the extent to which LDD probability and the log transformed vector seed load, respectively, vary between deer individuals and months. For this we used linear models with deer individual and sampling month as explanatory variables. We then regressed the log-transformed vector seed load against the logit-transformed LDD probability to determine whether they covary (Question 3). To test whether this relationship differs between plant species (Question 4), we fitted a linear model with the log-transformed vector seed load as the response variable and plant species, logit-transformed probability of LDD and their interaction as explanatory variables. This analysis was restricted to the 19 plant species which were dispersed by at least five different deer individuals. For the same species, we also investigated whether non-random associations between LDD probability and seed load affect the LDD potential of plant species (Question 5). To this end, we calculated the predicted LDD potential of a plant species as the mean of the per-seed LDD probabilities for each deer individual and month, weighted by the vector seed load of the respective plant species for that individual and month. We then compared this predicted LDD potential of each plant species to predictions of a null model in which we randomly permuted the plant species’ seed loads between both deer individuals and months. From 100,000 permutations, we calculated 95% bootstrap confidence intervals for the LDD potential of each plant species under random associations of LDD probability and seed load.

## Results

Dispersal simulations suggest that red deer are important LDD vectors (Fig. 2). Across all deer individuals and months, 20.7% of dispersal simulations led to a horizontal displacement greater than 1 km (99% quantile of horizontal displacement: 4.8 km). The predicted mean horizontal seed displacement by red deer (737 m) is somewhat greater than expected for non-flying mammals and birds of the same size (for a body mass of 67.1 kg an allometric study [63] predicts a dispersal distance of 498 m). Furthermore, 16.8% (16.9%) of dispersal simulations resulted in upward (downward) displacement greater than 50 m (1 and 99% quantiles of vertical displacement: − 278 m and 284 m, respectively).

### To what extent does the probability of LDD vary between individuals and months?

Predicted seed dispersal distances varied between individuals both in the horizontal and the vertical dimension (Fig. 2).

**Fig. 3:**
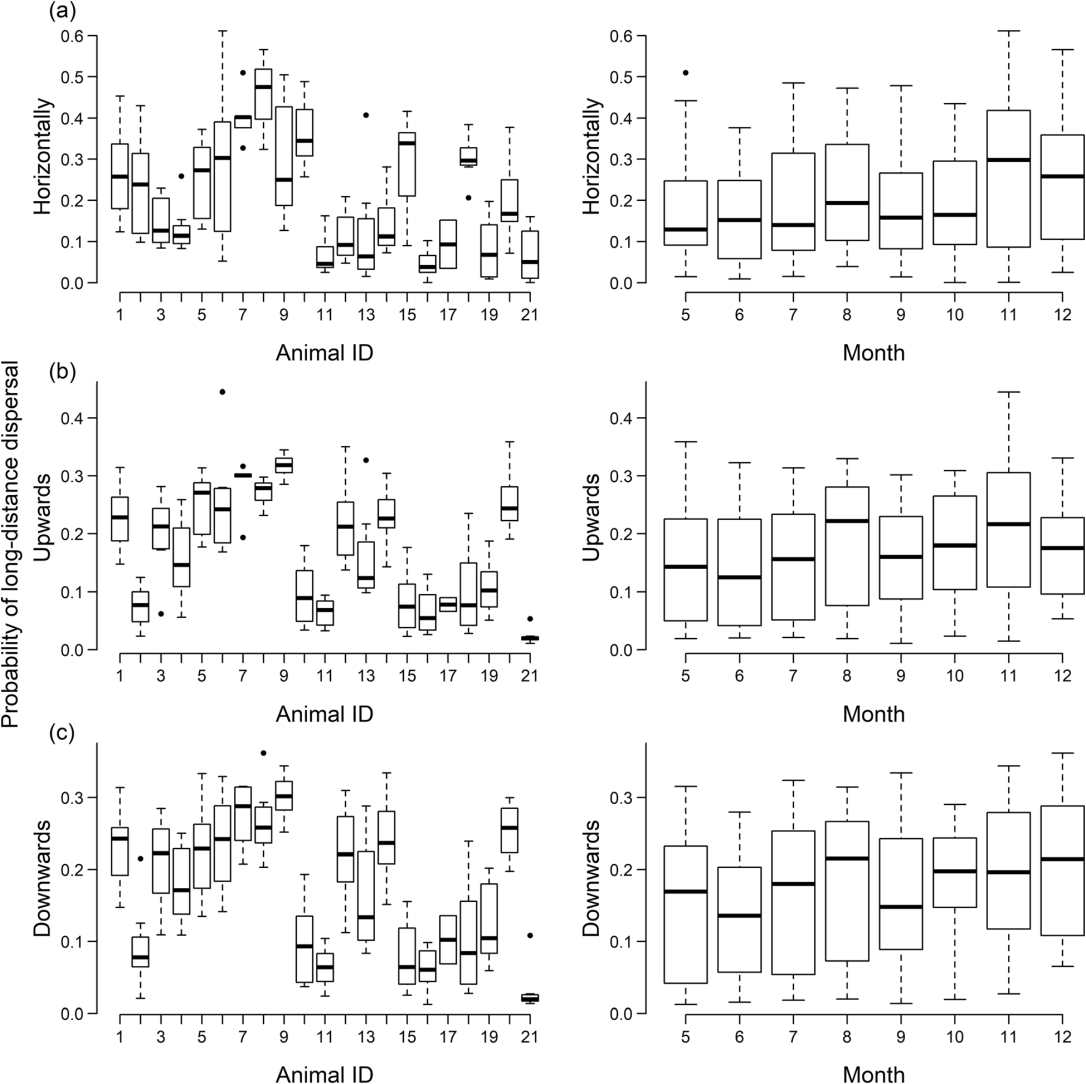
The variation in probability of long-distance dispersal between deer individuals (left column) and sampling month (right column) for each of horizontal (a), upward (b) and downward (c) dispersal respectively

LDD probability varied much more between deer individuals than between sampling months (Fig. 3). This held for horizontal LDD (> 1000 m) as well as for upward and downward LDD (> 50 m). Variation between deer individuals accounted for 63% of the variance in horizontal LDD probability (*F*_20, 137_ = 11.46; *p* < 0.001), 75% of the variance in upward LDD probability (*F*_20, 137_ = 20.01; *p* < 0.001) and 72% of the variance in downward LDD probability (*F*_20, 137_ = 17.83; *p* < 0.001). In contrast, sampling month only explained 6.7% (*F*_7,150_ = 1.54; *p* = 0.34), 4% (*F*_7,150_ = 0.9285; *p* = 0.49), and 6% (*F*_7, 150_, = 1.396; *p* = 0.21) respectively, of the variance in horizontal, upward and downward LDD probability.

To what extent does seed load vary between individuals and months?

Seedlings of at least 62 plant species emerged from the dung samples (see Table S1 in the Appendix). Seedling numbers per unit dung mass were then summed across all plant species to calculate the total seed load of a given animal in a given month.

**Fig. 4:**
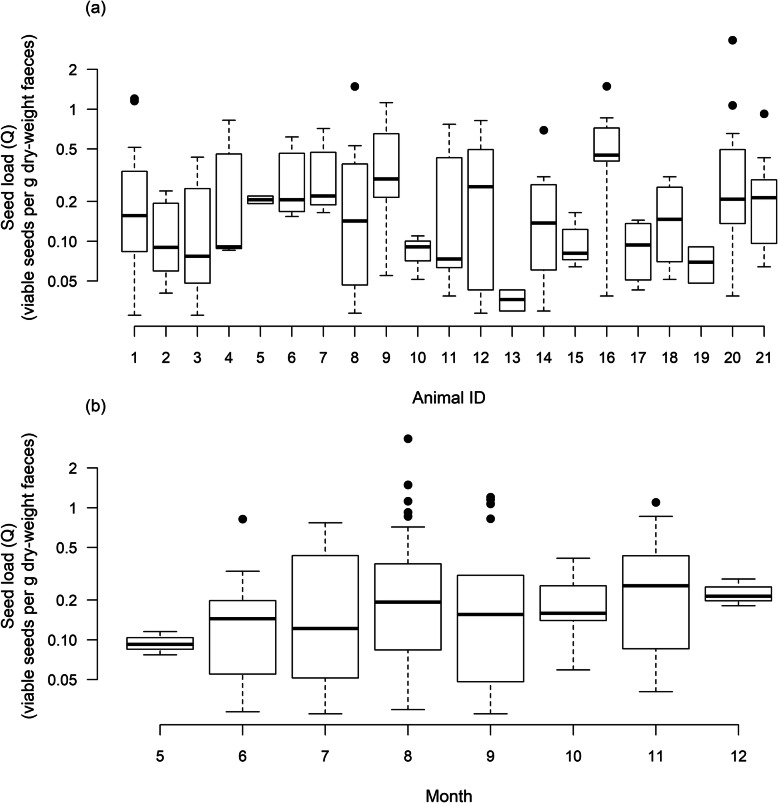
Variation in total seed load across individuals (a) and months (b) respectively. Total seed load is measured as the sum of viable seeds of all plant species per gram dry mass of faeces

Deer individual and month jointly explained 28% of the variance in log transformed vector seed load. Deer identity explained 23% of the variance in seed load (F20, 157 = 2.343, *p* < 0.01, Fig. 4a) whereas sampling month only explained 5% of the variance (F7, 170 = 1.314, *p* = 0.25, Fig. 4b).

**Fig. 5:**
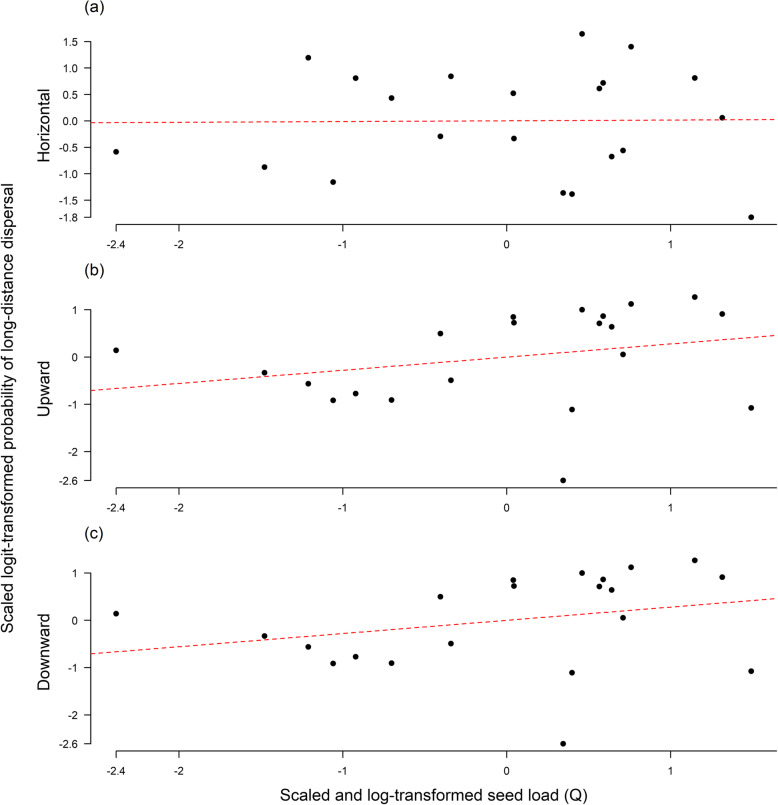
The relationship between the probability of long-distance dispersal (LDD) and seed load horizontally (a), upwards (b) and downwards (c). The dashed red lines indicate the predictions of the linear model, whilst the points indicate the 21 individual deer. In each case, prob. of LDD was scaled and logit transformed while seed load was scaled and log transformed. No significant correlations exist

Seed load did not account for significant variation in LDD probability in any of the three orientations considered (*F*_1, 19_ < 1.6, *p* > 0.22 for all three orientations) (Fig. 5).

**Fig. 6:**
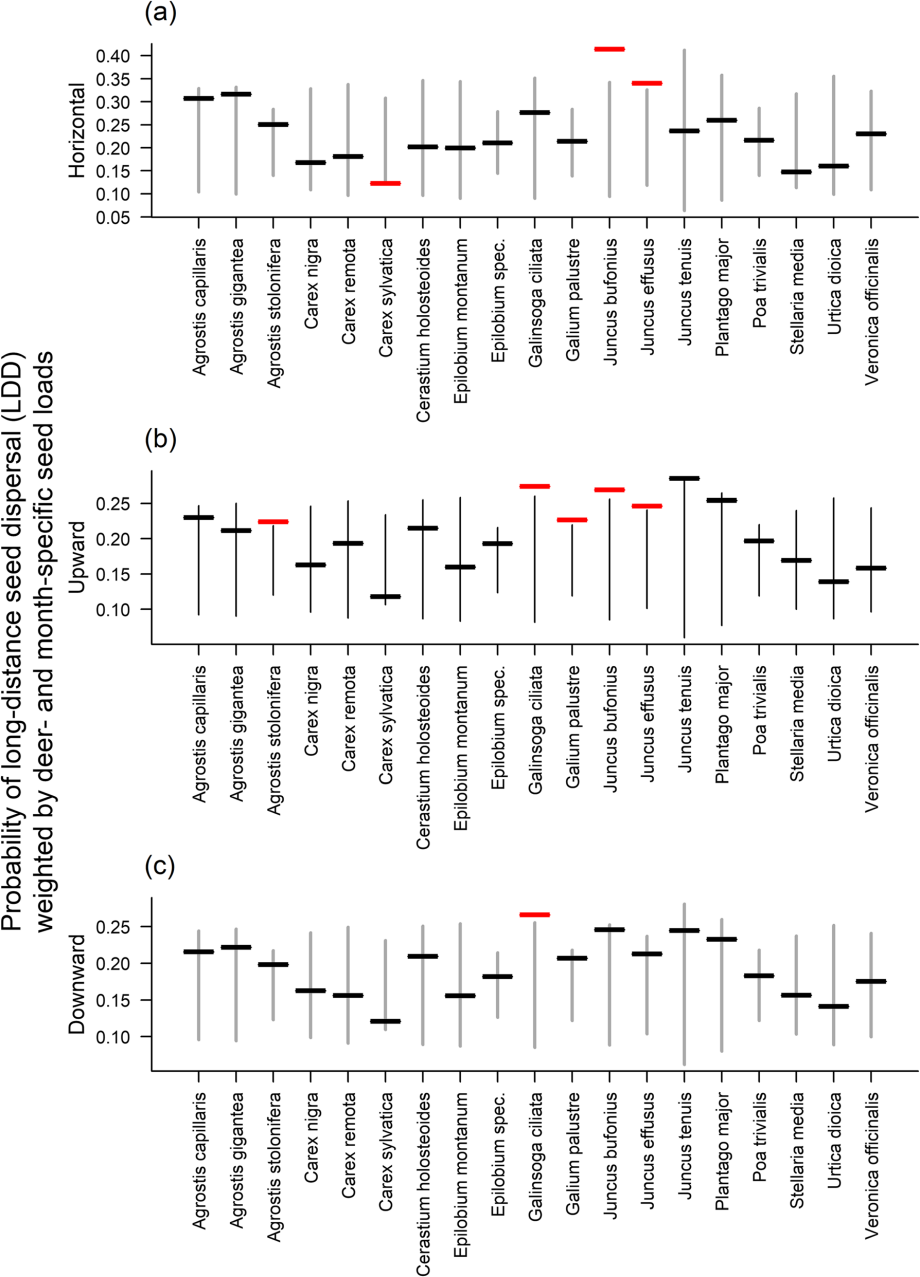
The predicted probability of long-distance seed dispersal (LDD) in the horizontal (a), upward (b) and downward (c) direction for 19 plant species found in the dung of at least 5 different deer individuals. Horizontal lines indicate predicted LDD probabilities that were obtained by weighting the per-seed LDD probability for individual deer and months with a plant species’ seed load for the respective deer and month. Vertical lines represent 95% confidence intervals of a null model in which per-seed LDD probability and seed load are randomly associated. Red horizontal lines show cases for which LDD probabilities deviate from these confidence intervals

Plant species did not differ significantly in their relationship between seed load and LDD probability in any of the three orientations (*Partial* − *R*^2^ < 0.17, *F*_(144,18)_ < 1.4, *p* > 0.05 for all three orientations – see Figs. S2, 3, 4). Nevertheless, when we weighted the predicted per-seed LDD probability for each deer individual and month by the seed load of particular plant species for these individuals and months, we found that the predicted LDD potential varied notably between plant species: horizontal LDD potential varied 3.4-fold (range: 0.12–0.41, Fig. 6a), upward LDD potential varied 2.4-fold (range: 0.12–0.29, Fig. 6b) and downward LDD potential varied 2.2-fold (range: 0.12–0.27, Fig. 6c). For three of 19 plant species, the predicted potential for horizontal LDD deviated from the 95% confidence interval of a null model that randomly associates per-seed LDD probability and seed load (Fig. 6a). Upward LDD potential deviated from the 95% confidence interval for five species (Fig. 6b), whereas downward LDD potential deviated for one species (Fig. 6c). The observed number of nine deviations from the 95% confidence intervals is very unlikely under the null model (across-species *p* = 0.002 calculated as the probability of obtaining ≥ 9 deviations under a binomial distribution with success probability 0.05 and sample size 57 = 3 × 19). In eight of these nine cases, the non-random associations between seed load and per-seed LDD probability increased LDD potential (Fig. 6).

## Discussion

Our study shows that variation in the movement and feeding behaviour of red deer individuals has profound effects on the long-distance seed dispersal (LDD) of a variety of plant species. We found that the proportion of extreme displacement events (horizontal, upward and downward per-seed LDD probability) varied substantially between deer individuals but showed little seasonal variation (Figs. 3 and 4). This large variation between individuals is even more remarkable given that all studied individuals belonged to a narrow subset of the deer population (breeding-age females).

Seed load (as the dispersal-relevant outcome of feeding behaviour) was not significantly affected by seasonal variation (Fig. 4b), a surprising result given the strong phenology of plant species in the study region, which mostly fruit in summer and autumn [64]. Significant variation in seed load was, however, observed between individuals (Fig. 4a). The drivers of this variation are unclear. It could for example be that certain deer individuals simply consume more seeds than others or that they favour microhabitats, plant species or plant individuals with a high number of seeds suitable for endozoochory. Other possible explanations could be differences in seed predation rates or the quality of gut treatment.

Inter-individual differences in LDD probability and overall seed load were not correlated with each other (Fig. 5), indicating that they independently act on LDD, neither reinforcing nor weakening differences in the LDD services of individual deer. The relationship between LDD probability and seed load also did not differ significantly between plant species (Fig. S2, 3, 4). Still, plant species differ in how their seed load is distributed across deer individuals and months and this leads to considerable interspecific variation in LDD potential (Fig. 6). Moreover, for a substantial number of plant species we found non-random associations between seed load and LDD probability. Almost all of these non-random associations concentrated seed load on more mobile deer, thereby promoting LDD (notably upward LDD) of the respective plant species (Fig. 6). This study cannot identify the mechanisms that cause such non-random associations and the mechanisms that cause deer individuals to differ in per-seed LDD probability and overall seed load. To further progress in understanding seed dispersal by red deer, it will be necessary to replace stochastic descriptions of these differences by deterministic descriptions of processes [65]. In particular, it is important to understand which factors cause deer individuals to differ in movement behaviour (determining LDD probability) and feeding behaviour (determining both overall seed load and seed load of individual plant species).

Predictable differences in the dispersal services of individual deer could depend on properties of individuals, properties of the environments they inhabit, or some combination of the two. Tucker et al. (2018) [66] suggest for example that animal movement is reduced in environments that are more heavily impacted by humans. Their argument is, however, based on the Human Footprint Index which is unlikely to play a major role within the park borders. ‘Landscapes of Fear’, an increasingly popular term used to reference the “spatially explicit distribution of perceived predation risk” [67] represents another possible explanation for the observed differences [68]. From the perspective of red deer, the primary sources of fear within the study area could be considered to be wolf (*Canis lupus*), lynx (*Lynx lynx*), human hunting and human tourism. Wolves are only present in low numbers within the park, making them unlikely to drive the observed differences (although it is conceivable that even the scent of rare wolves could shape landcsapes of fear). Lynx are known to prey predominantly on roe deer [69] and hunting by humans occurs only in the buffer zone, meaning that these factors are also likely to play a marginal role at most. Tourism could be a significant driver of fear in the study area, although this would follow temporally well defined spikes of relatively low duration (primarily long-weekends) which are unlikely to explain the level of individual variation observed. Habitat effects have also been shown to affect the behaviour of various species [70, 71] with forest cover found to be a significant factor affecting the distribution of both red and roe deer in the bavarian forest [52]. Habitat distribution is, however, largely homogenous across the park and hence unlikely to explain the individual variation observed.

On the other hand, differences could well be driven by individual properties, in which case questions arise as to whether these differences are temporally variable (e.g. effects of ontogeny or health) or constant throughout the lifetime of individuals (e.g. genetic effects or behavioural syndromes). Since all studied individuals fell within the same general age bracket, ontogeny is not thought to have played a significant role here, and we have no reason to believe that any of the studied individuals suffered from health issues. Behavioural syndromes as manifest in distinct behavioural phenotypes (behavioural types) [34] offer a promising explanation. Behavioural types are the manifestations of different personality traits, normally measured on five axes (viz. boldness, aggressiveness, activity, exploratory behaviour, and sociability) [72]. Such personality traits are difficult to measure on large wild animals and thus Hertel et al. (2019) used remote tracking data in an attempt to quantify behavioural syndromes in brown bear (*Ursus arctos*) [73, 74]. They showed that tracking devices can be highly useful in assessing behavioural repeatability, but that interpreting these behaviours along the classical five axes is not straightforward [74]. Such an approach could be applied to the movement data collected here in order to study effects of behavioural variation on LDD services provided by individual deer. This is certainly worth further investigation since knowing more about the behavioural syndromes of a seed dispersal vector (such as red deer), and how these affect its dispersal service, would allow the more accurate parameterization of models predicting overall seed dispersal at the population or landscape level. In turn, this may provide valuable input for policy decisions such as the setting of hunting quotas or the designation of protected area boundaries.

It will be exciting to study whether further determinants of seed dispersal vary between individual animal vectors. In addition to the individual variation in movement and seed load per unit dung mass demonstrated here, one could test for individual differences in seed passage time and dung volume. At the interspecific level, there is indeed evidence that displacement velocity, seed passage time and seed load per animal all increase with the body mass of the animal [22]. Still, it is questionable whether these allometric relationships play an important role for our study population in which body mass varies much less than in interspecific comparative analyses. Finally, this study has largely focused on the quantity component of seed disperser effectiveness rather than the quality component (the probability that a dispersed seed produces a new recruit) [75]. It should be noted, however, that by distinguishing between upward and downward dispersal, we considered one important aspect of the quality component: under climate change upward dispersal is likely to result in higher recruitment than downward dispersal [62]. Taking into account further components of disperser quality (such as the probability of seed survival in the gut and the microsites in which seeds are deposited) holds perhaps the greatest potential to advance the understanding of dispersal services provided by red deer.

For future studies choosing to incorporate inter-individual variation into their assessments, assignment error (assigning dung to the wrong individual) is a notable challenge to be considered. We minimised this by studying geographically isolated individuals and minimising the time lag between a location fix and sample collection. Since fresh samples are easily distinguishable from older ones by their mucous layer and the dung of adult hinds is readily distinguishable from that of their offspring, we expect assignment errors to be small. Future studies could further reduce assignment errors by genotyping animals and the mucous layer around dung pellets. The benefits of this approach must, however, be weighed against the increased financial and time demands.

The observed high variance in LDD services of individual animals substantially reduces the reliability of LDD provided by the entire population. This reduced reliability stems from the fact that the total LDD service of an animal population becomes more variable as the inter-individual variance increases. In analogy to demographic stochasticity (which is more important in small populations [76]), the reliability of LDD will be particularly low when LDD depends on a small population of seed-dispersing animals with high inter-individual variance in LDD service. In this case, the overall LDD service of the population critically depends on whether a few individuals with high mobility and/or seed load are present. Hence, quantifying the magnitude of inter-individual variance in LDD service is important to assess how declines in the population size of key seed-dispersing animals will propagate to plant communities.

## Conclusions

We found that inter-individual variation in the movement and feeding behaviour of red deer has a profound effect on LDD of a variety of plant species. Plant species varied more than twofold in LDD potential because they differed in how seed load was distributed differently across deer individuals and in time. Notably, for several plant species we detected increased LDD potential which arises since their seed load is non-randomly concentrated on deer individuals with high per-seed LDD probability. More generally, the observed high variance in LDD service between deer individuals reduces the reliability of the total LDD service provided by the deer population, especially when this population is small. Studies projecting LDD from species-level averages of movement and feeding behaviour overestimate the reliability of LDD and underestimate the consequences that population declines of key seed-dispersing animals have for the LDD of multiple plant species. Since LDD is of fundamental importance to core ecological processes and the preservation of biodiversity in a changing world, we propose that such inter-individual variation in vector behaviour be incorporated into quantitative assessments of LDD.

## Supplementary information

**Additional file 1: Figure S1.** Simulated distribution of seed passage times based on the statistical model of Picard et al (2015)

**Additional file 2: Table S1.** Frequency of plant species emerging from dung samples of the studied deer individuals.

**Additional file 3: Figure S2.** The relationship between scaled and logit transformed probability of long-distance horizontal dispersal and scaled and log-transformed seed load. Pearson correlation coefficient is given in brackets; bold indicates a significant correlation.

**Additional file 4: Figure S3.** The relationship between scaled and logit transformed probability of long-distance upward dispersal and scaled and log-transformed seed load. Pearson correlation coefficient is given in brackets; bold indicates a significant correlation

**Additional file 5: Figure S4.** The relationship between scaled and logit transformed probability of long-distance downward dispersal and scaled and log-transformed seed load. Pearson correlation coefficient is given in brackets; bold indicates a significant correlation

## Data Availability

The data generated from the greenhouse seedling emergence study are included in this published article [and its supplementary information files]. Additional data concerning the greenhouse experiment are available upon reasonable request. The animal movement datasets analysed during the current study are not publicly available due the risk of them being used for poaching. They can be made available from the corresponding author on reasonable and verifiable request.

## Abbreviations

3D: Three dimensional
BFNP: Bavarian Forest National Park
GPS: Geographical Positioning System
GSM: Global System for Mobile Communications
ITV: Intraspecific Trait Variability
LDD: Long Distance Dispersal
UTM: Universal Transverse Mercator
VHF: Very High Frequency
