# Supplementary figures and images for: The importance of individual movement and feeding behaviour for long-distance seed dispersal by red deer: a data-driven model
Source: Mov Ecol. 2020 Oct 28;8:44. doi: 10.1186/s40462-020-00227-5 (PMC7594291; doi:10.1186/s40462-020-00227-5)

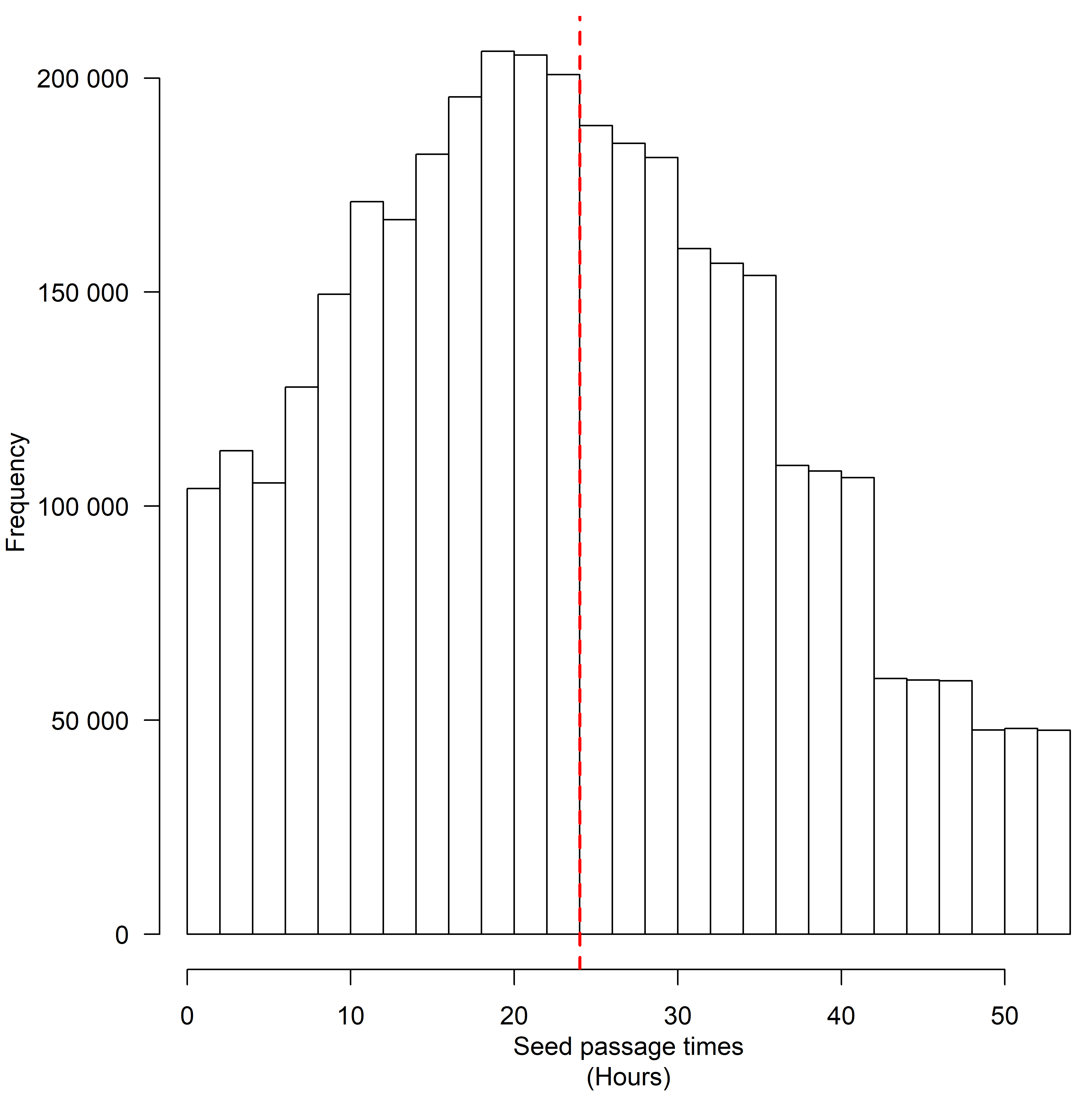

Supplement: Supplementary file 1 — Additional file 1: Figure S1. Simulated distribution of seed passage times based on the statistical model of Picard et al (2015) [file 40462_2020_227_MOESM1_ESM.tiff]

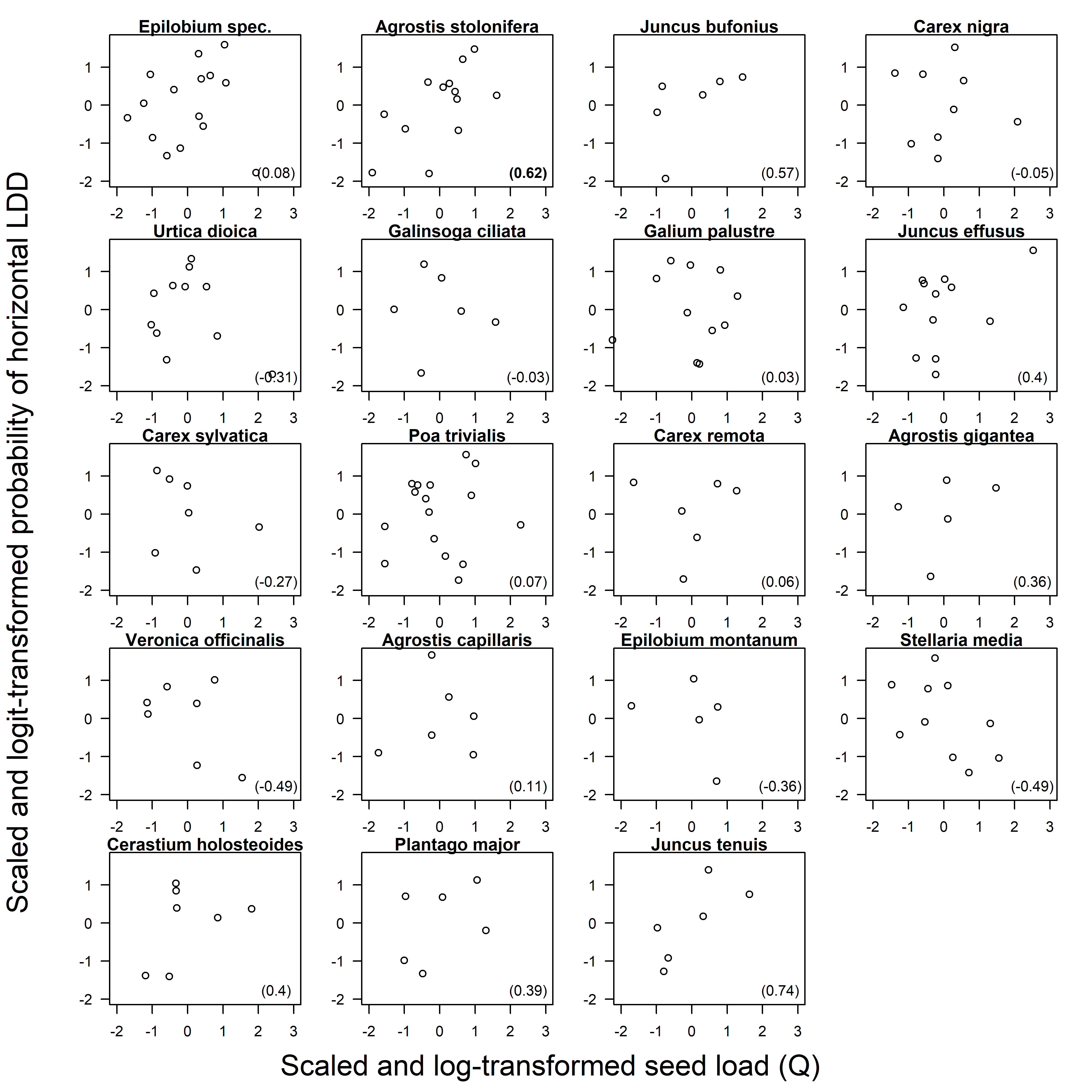

Supplement: Supplementary file 3 — Additional file 3: Figure S2. The relationship between scaled and logit transformed probability of long-distance horizontal dispersal and scaled and log-transformed seed load. Pearson correlation coefficient is given in brackets; bold indicates a significant correlation. [file 40462_2020_227_MOESM3_ESM.tiff]

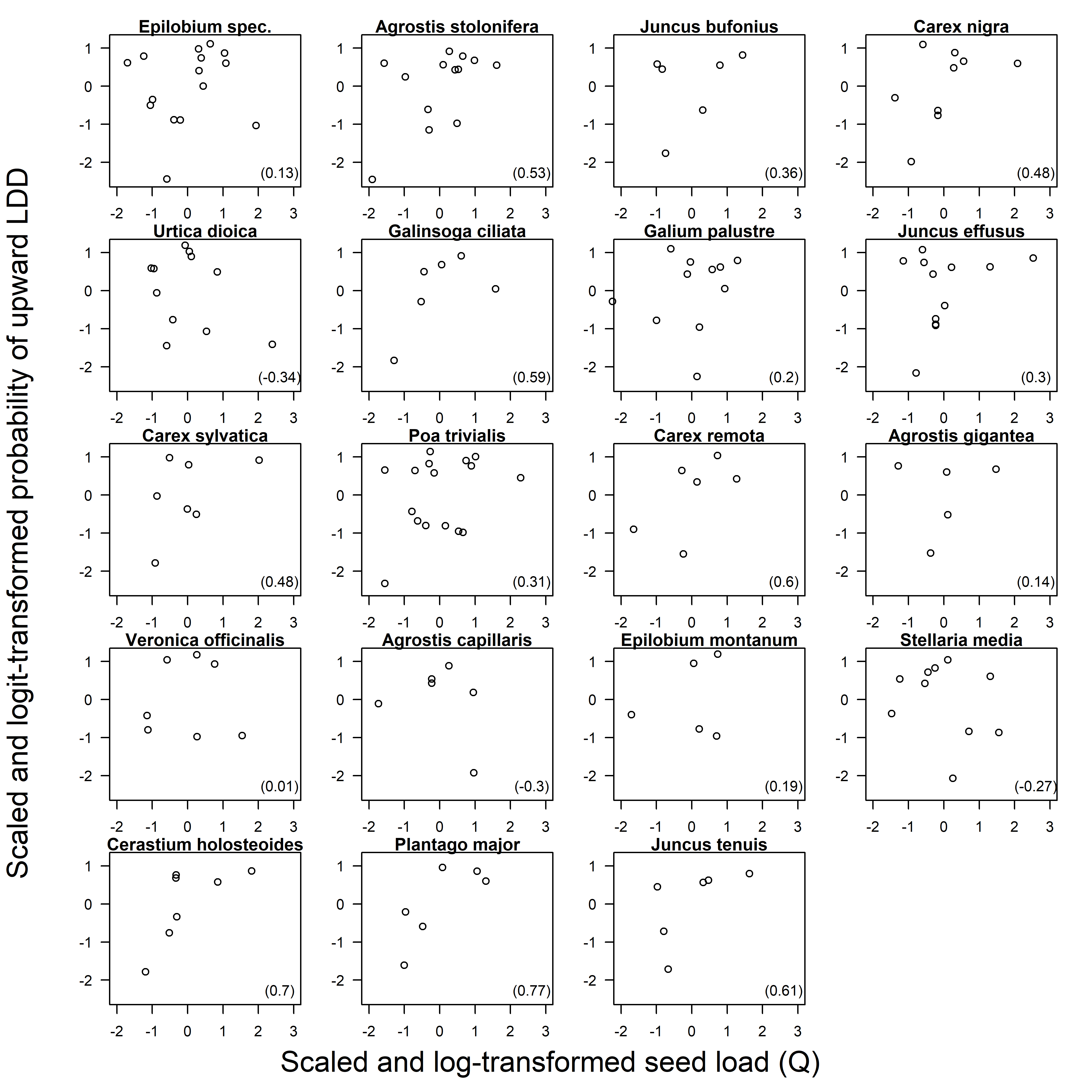

Supplement: Supplementary file 4 — Additional file 4: Figure S3. The relationship between scaled and logit transformed probability of long-distance upward dispersal and scaled and log-transformed seed load. Pearson correlation coefficient is given in brackets; bold indicates a significant correlation [file 40462_2020_227_MOESM4_ESM.tiff]

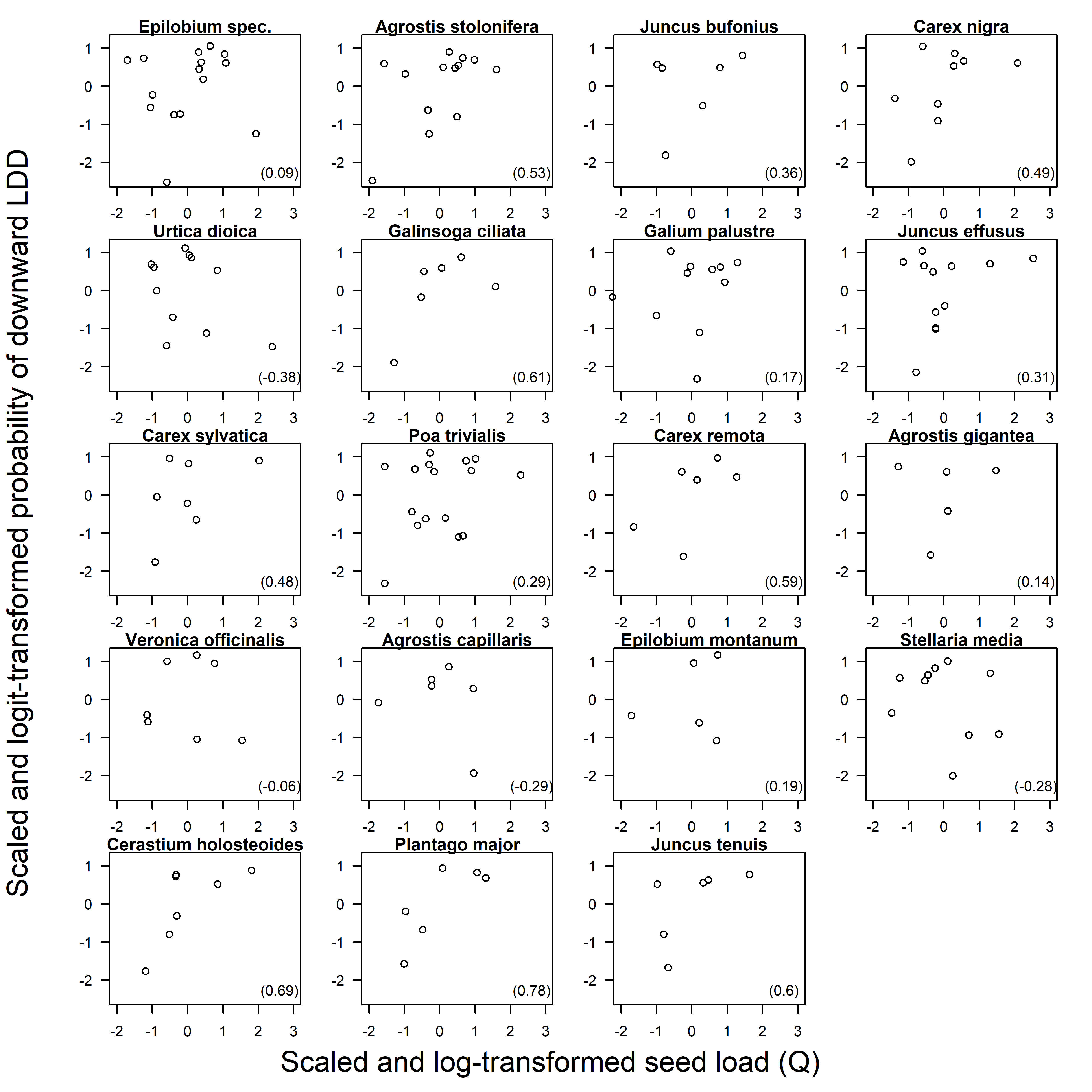

Supplement: Supplementary file 5 — Additional file 5: Figure S4. The relationship between scaled and logit transformed probability of long-distance downward dispersal and scaled and log-transformed seed load. Pearson correlation coefficient is given in brackets; bold indicates a significant correlation [file 40462_2020_227_MOESM5_ESM.tiff]
