# Supplementary material for: The importance of individual movement and feeding behaviour for long-distance seed dispersal by red deer: a data-driven model
Source: Mov Ecol. 2020 Oct 28;8:44. doi: 10.1186/s40462-020-00227-5 (PMC7594291; doi:10.1186/s40462-020-00227-5)
Supplement: Supplementary file 2 — Additional file 2: Table S1. Frequency of plant species emerging from dung samples of the studied deer individuals. [file 40462_2020_227_MOESM2_ESM.docx]

**Table S1 – Frequency of plant species emerging from dung samples of the studied deer individuals.**

| **Species** | **Number of seedlings** |  | **Species** | **Number of seedlings** |
| --- | --- | --- | --- | --- |
| *Agrostis canina* | 1 |  | *Juncus spec.* | 3 |
| *Agrostis capillaris* | 22 |  | *Juncus tenuis* | 45 |
| *Agrostis gigantea* | 9 |  | *Leontodon autumnalis* | 2 |
| *Agrostis* sp*.* | 1 |  | *Lolium perenne* | 4 |
| *Agrostis stolonifera* | 29 |  | *Luzula pilosa* | 1 |
| *Alopecurus aequalis* | 2 |  | *Luzula sylvatica* | 3 |
| *Ayuga reptans* | 1 |  | *Lycopersicum esculentum* | 1 |
| *Capsella bursa-pastoris* | 2 |  | *Lysimachia nummularia* | 8 |
| *Cardamine pratensis* | 1 |  | *Lysimachia volgaris* | 1 |
| *Carex canescens* | 2 |  | *Myosotis palustris* | 1 |
| *Carex flacca* | 3 |  | *Plantago lanceolata* | 2 |
| *Carex moorcroftii* | 1 |  | *Plantago major* | 53 |
| *Carex nigra* | 20 |  | *Poa palustris* | 1 |
| *Carex panicea* | 2 |  | *Poa pratensis* | 3 |
| *Carex remota* | 12 |  | *Poa trivialis* | 53 |
| *Carex spec.* | 6 |  | *Potentilla supina* | 1 |
| *Carex sylvatica* | 24 |  | *Prunus spec.* | 1 |
| *Cerastium holosteoides* | 18 |  | *Rumex acetosa* | 2 |
| *Chenopodium album* | 21 |  | *Sagina procumbens* | 10 |
| *Deschampsia cespitosa* | 5 |  | *Senecio sylvaticus* | 1 |
| *Digitaria sanguinalis* | 1 |  | *Sonchus arvensis* | 4 |
| *Epilobium montanum* | 12 |  | *Stellaria alsine* | 3 |
| *Epilobium spec.* | 98 |  | *Stellaria media* | 29 |
| *Festuca rubra* | 4 |  | *Trifolium repens* | 5 |
| *Galinsoga ciliata* | 16 |  | *Urtica dioica* | 76 |
| *Galium palustre* | 521 |  | *Verbascum nigrum* | 1 |
| *Hieracium sylvaticum* | 1 |  | *Verbascum thapsus* | 1 |
| *Hypericum humifusum* | 1 |  | *Veronica beccabunga* | 2 |
| *Impatiens spec.* | 1 |  | *Veronica montana* | 1 |
| *Juncus bufonius* | 15 |  | *Veronica officinalis* | 30 |
| *Juncus conglomeratus* | 7 |  | *Veronica serpyllifolia* | 10 |
| *Juncus effusus* | 64 |  | Total | 1281 |
